# Hospital Festivals and Meetings

**Published:** 1894-08-04

**Authors:** 


### HOSPITAL FESTIVALS AND MEETINGS.

### HOSPITAL SUNDAY FUND.

The council meeting of the Metropolitan Hospital Sunday Fund was held on Monday, July 30th, at the Mansion House, to receive the report of the Committee of Distribution. The Lord Mayor, the president and treasurer, was in the chair, and amongst those present were Sir Sydney Waterlow, Sir Owen Roberts, Sir Saville Crossley, Mr. H. Cosmo Bonsor, M.P., Archdeacon Sinclair, and Mr. H. N. Custance (secretary).

Sir Sydney Waterlow announced that the sum available for distribution this year was £41,800, as against £35,400 last year, and the committee were able to propose that of this £39,865 should be divided amongst 127 hospitals and 55 dispensaries, as against £35,056 in 1893. Five per cent. (about £2,000) is set aside to purchase surgical appliances in monthly proportions during the next twelve months. The greater success of the Fund this year has been largely due to the munificent gift from Sir Saville Crossley—ever a generous contributor. Sir Sydney Waterlow went on to say that the committee recommended no award in the case of a hospital against which certain charges had recently been made. If the institution in question were reconstructed on a satisfactory basis, and applied for a grant next year, it would be judged upon its merits. He was glad to find that the entire staff were leaving, and trusted better things might be now looked for in connection with it. It was the first time during the 22 years he had sat on the committee such a scandalous case had come before them.

The Rev. J. F. Mills seconded the motion, which was adopted.

